# Experimental folate deficiency in human subjects: what is the influence of vitamin C status on time taken to develop megaloblastic anaemia?

**DOI:** 10.1186/s12878-018-0107-2

**Published:** 2018-06-19

**Authors:** Paul Henry Golding

**Affiliations:** Unit 10, Laurel Springs, 18 Doolan Street, Nambour, QLD 4560 Australia

**Keywords:** Experimental folate deficiency, Megaloblastic anaemia, Liver folate, Folate kinetics and metabolism, Self-experimentation, Vitamin C deficiency

## Abstract

**Background:**

In 1962 Victor Herbert developed megaloblastic anaemia four months after commencing a severely folate-deficient diet whereas, in his self-experiment 50 years later, this author took 19 months to fully deplete his liver folate store. This author proposed that his own larger initial liver folate store, due to his vegetarian diet and consumption of fortified foods, was the cause of the time difference.

**Main text:**

This author now proposes that Herbert was also likely to have been deficient in vitamin C, thus shortening the time taken to develop folate deficiency. Several human experiments have confirmed the role of vitamin C in protecting reduced forms of folate from oxidation. Although there has historically been no consensus on the required intake of vitamin C, and official recommendations set a level below that required to ensure plasma saturation, recent research supports an intake that would ensure saturation. There have been no longitudinal experiments on human subjects since the introduction of voluntary or mandatory folic acid fortification of food, and the few published models differ significantly in their estimates of human liver folate storage capacity.

**Conclusion:**

Because of the importance of folate in one-carbon metabolism, the potential influence of vitamin C intake on the time taken to deplete the liver folate store should be experimentally investigated.

**Electronic supplementary material:**

The online version of this article (10.1186/s12878-018-0107-2) contains supplementary material, which is available to authorized users.

## Background

**Fig. 1:**
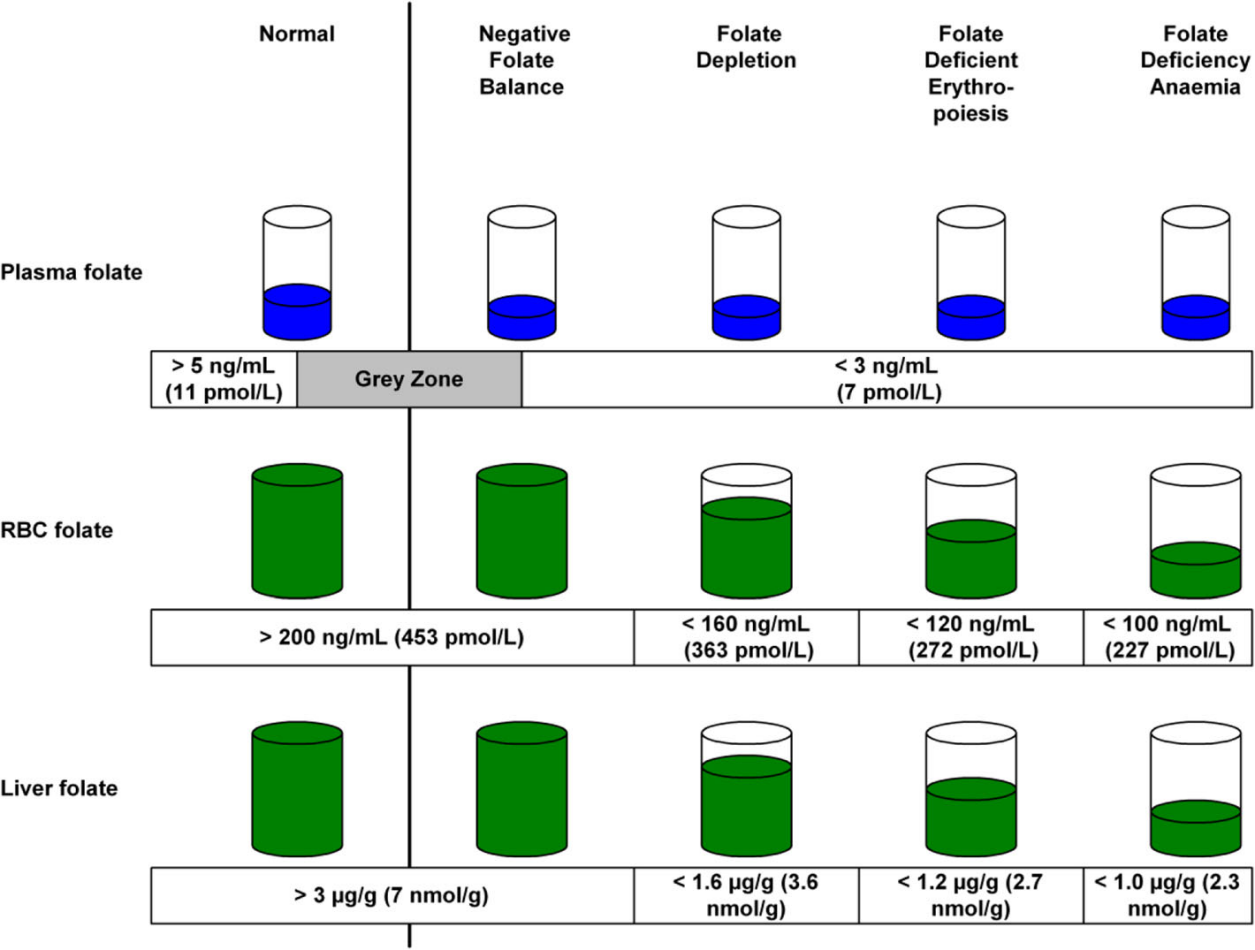
Stages in the development of folate deficiency. Derived from Herbert [2]

Based on his pioneering self-experiment [1], Victor Herbert proposed a model for the *Sequential stages in the development of folate deficiency* [2]. According to this model, folate deficiency develops in a sequence of four distinct stages, with changes in specific biochemical and haematological markers defining the borders between them (Fig. 1).

In the first stage, the earliest sign of folate deficiency is marked by the change from *Normal* to *Negative Folate Balance*, with serum folate concentration falling from > 11 pmol/L to < 7 pmol/L. The second stage, *Folate Depletion*, commences when red-cell folate falls from > 453 pmol/L to < 363 pmol/L. Haematology is normal for the first two stages. The third stage, *Folate Deficient Erythropoiesis*, is marked by the commencement of the initial abnormal haematology in the form of neutrophil hypersegmentation. The fourth stage, *Folate Deficiency Anaemia*, is marked by overtly abnormal haematology in the form of neutrophil hypersegmentation, macroovalocytes, elevated MCV and low haemoglobin.

**Fig. 2:**
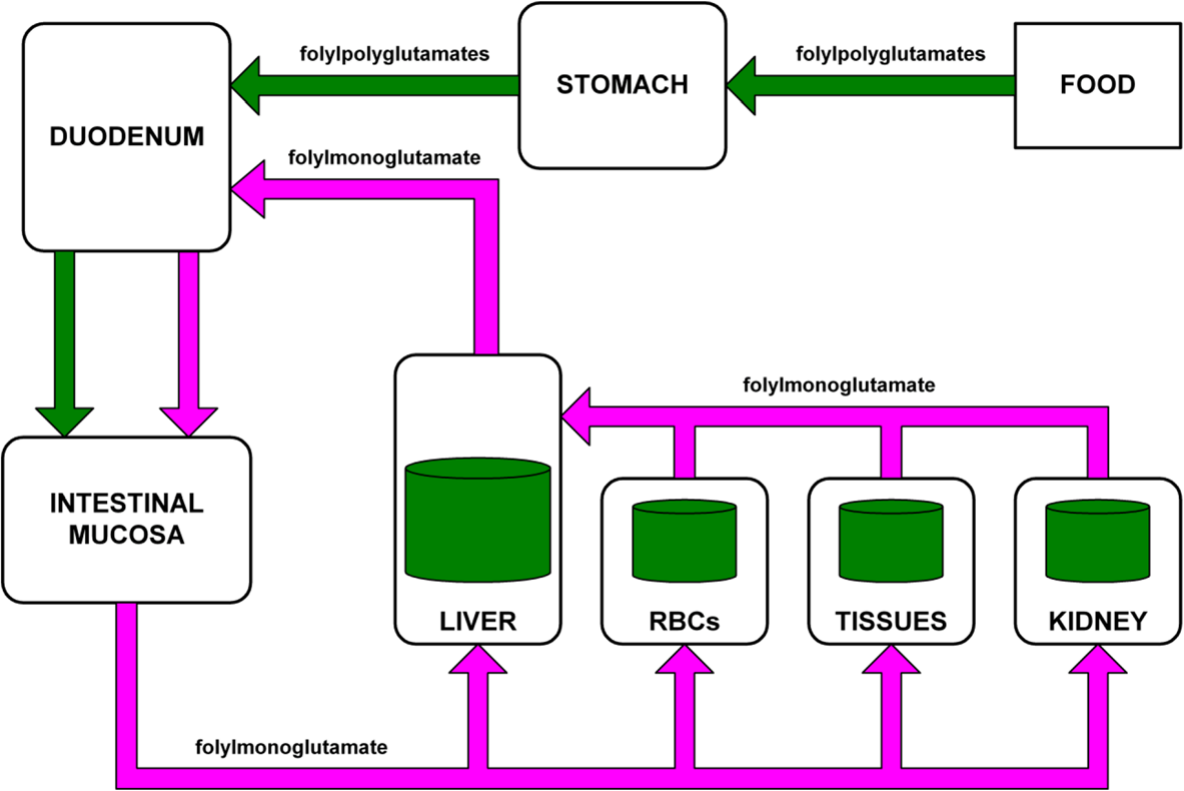
Enterohepatic recycling of folate. Derived from Steinberg [4]. Folylpolyglutamates are the storage form of folate, and folylmonoglutamate is the transport form [46]. The food folate, in the form of folylpolyglutamates, is converted to folylmonoglutamate in the intestinal mucosa then transported in the blood plasma to the liver, red-cells, tissues and kidneys where it is converted back to the folylpolyglutamate form for storage or metabolism. When required, the stored folylpolyglutamates may then be converted back to folylmonoglutamate and transported to the liver where it may be recycled, via the bile

Herbert developed megaloblastic anaemia four months after commencing a folate-deficient diet [1]. Herbert [3] states that the life-span of red cells, and the time taken to deplete the human liver folate store, are coincidentally both four months. This is problematic because, if Herbert’s liver had initially contained a four-month supply of folate and if his red-cell folate was initially normal, he should not have developed megaloblastic anaemia of folate deficiency after only four months. Enterohepatic recycling of folate (Fig. 2), including folate scavenged from senescent red cells, should have allowed the liver to continue to supply folate to rapidly reproducing cells beyond the lifetime of the first generation of red cells [4, 5].

As noted by Lindenbaum et al. [6] and Stabler [7], the few longitudinal experiments of Herbert’s era were affected by several known confounding factors. Ethical considerations have prevented further experimentation to induce folate deficiency in healthy volunteers. Several attempts have since been made to model the development of folate deficiency [5, 8, 9]. Gregory [9] and Lin [5] noted the very marked differences between models for folate storage capacity and, as stated by Lin, these discrepancies need to be investigated experimentally.

**Fig. 3:**
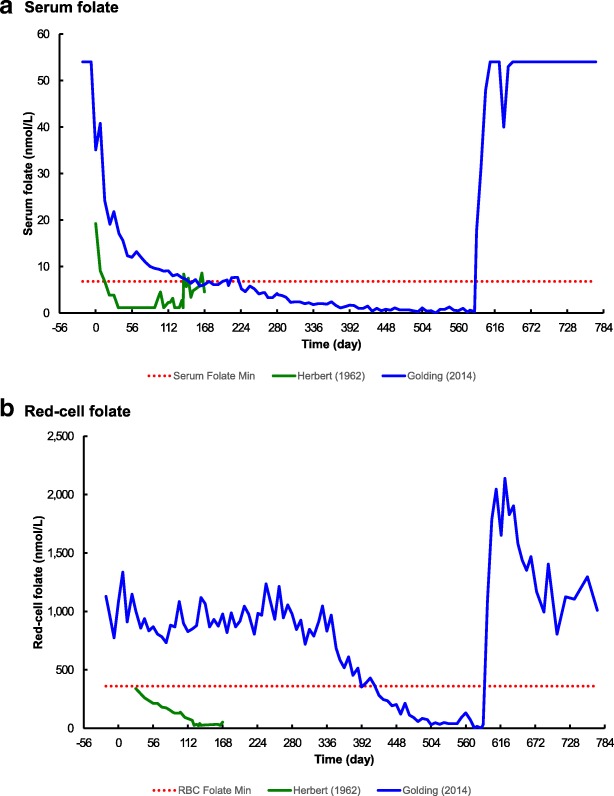
Comparison of times taken to develop folate deficiency. Derived from Herbert [1] and Golding [10]. **a** Serum folate versus time. **b** Red-cell folate versus time. The dotted lines in **a** and **b** are the minimum normal values according to Bates and Lewis [47]

In his own longitudinal self-experiment, fifty years after Herbert, this author took 19 months to fully deplete his liver folate store [10]; this result is consistent with Lin’s model but inconsistent with Herbert’s result (Fig. 3). This author then suggested that the difference in time was caused by his consumption of folate fortified food, for many years prior to commencing the experiment, and therefore had a much larger initial liver folate store. This author now additionally proposes that there was a significant previously unknown confounding factor in Herbert’s experiment; his liver folate store was rapidly depleted because of a vitamin C deficiency.

Current information about human folate storage capacity continues to be based on Herbert’s experiment; for example, Hoffbrand and Provan [11] continue to state that we store only a four-month supply of folate. Is this appropriate in the context of voluntary or mandatory folic acid fortification in several countries, and the possibility of Herbert’s experiment being confounded by vitamin C deficiency? Should there be further experiments to investigate the time taken to deplete human folate stores, under different conditions of vitamin C status and initial liver folate levels?

This article examines the evidence for Herbert having been affected vitamin C deficiency, and its potential influence on the time taken to deplete the folate store.

## Main text

### Vitamin C status and folate deficiency

As noted by Brown [12] in 1951, scurvy is often accompanied by anaemia, and this may be microcytic, normocytic or macrocytic. Brown studied 46 patients, mostly older males on poor diets almost devoid of vegetables, with interventions including vitamin C, improved diet and bed rest. Based on his own experimental results, and other published studies in which the anaemia was successfully treated with vitamin C, he concluded that vitamin C has some influence on the production of red cells. Brown also concluded that, because of the variations in the type of anaemia observed in the malnourished subjects, it was likely that there were several aetiologies. In 1955, Brown [13] reported on a case in which the macrocytic anaemia and scurvy were both successfully treated with only vitamin C. It was not known, until Herbert’s experiment [1], that macrocytic anaemia may be caused by a dietary folate deficiency.

In 1955, Mueller and Will [14] reviewed the involvement of folate, vitamin B_12_ and vitamin C in the development of megaloblastic anaemia. This review, based on a series of experiments on humans performed in their own laboratory over a period of eight years, presented a hypothesis detailing the proposed interrelationship between the three vitamins. One proposal was that folate deficiency may occur secondary to a vitamin B_12_ deficiency; this is now known as the “folate trap” hypothesis. Another proposal was that vitamin C is somehow necessary for folate and vitamin B_12_ metabolism; this was based on their finding that 48 subjects with macrocytic anaemia had lower plasma vitamin C concentrations than a control group on a similar diet.

In the 1960s, there were several reported cases in which megaloblastic anaemia, concurrent with scurvy, responded to treatment with vitamin C alone. Will and Murdoch [15] described three such cases and noted that vitamin B_12_ concentrations were normal. They suggested that, where there is a deficiency of dietary vitamin C, there might also be a deficiency of dietary folate but, because this was before Herbert’s experiment [1], they did not know the dietary folate requirement. Cox et al. [16] reported that, in five out of six cases of megaloblastic anaemia, treatment with vitamin C alone resolved the haematological abnormalities. Asquith et al. [17] also reported such a case; they discussed several other similar reported cases, noting that there had been no increase in folic acid intake during successful treatment for megaloblastic anaemia with vitamin C. They noted that Zalusky and Herbert [18] had found no response to vitamin C alone and suggested that this might be due to the patient having a more severe folate deficiency.

In their 1961 report, Zalusky and Herbert [18] described a case in which a patient with concurrent scurvy and megaloblastic anaemia showed no haematological response to large doses of vitamin C alone. Although the patient was severely malnourished, with no measurable dietary folate and severe folate deficiency evidenced by blood and bone marrow abnormalities, serum vitamin B_12_ was normal. While maintained on a high-energy diet devoid of folate, the haematological abnormalities continued to worsen; this continued, even after 1 g/day intravenous ascorbic acid was commenced, with overt megaloblastic changes and accelerating haematological deterioration. The patient’s condition rapidly improved, returning to normal haematology, after treatment with 50 μg intramuscular folic acid daily. Zalusky and Herbert noted the difference between this case and reports of others where vitamin C alone was sufficient and suggested that the treatment diets of those patients might not have actually been devoid of folic acid. They proposed that the effect of vitamin C was to protect the reduced folates from oxidation, rather than it having a direct haematological role.

In 1975, Stokes et al. [19] reported on a case in which they successfully treated megaloblastic anaemia in a man with concurrent scurvy, using oral folinic acid (5-formyl-THF, 5-formyltetrahydrofolate) and vitamin C. Before treatment commenced, there was zero vitamin C in the plasma; the folate in the urine was mostly 10-formylfolic acid. After treatment with the folinic acid and vitamin C, the folate in the urine was mostly 5-methyl-THF (5-methyltetrahydrofolate), with no 10-formylfolic acid present. They concluded that, in the absence of vitamin C, the metabolically active l0-formyl-THF (10-formyltetrahydrofolate) was irreversibly oxidised to the inactive10-formylfolic acid. They therefore postulated that vitamin C protects reduced folates from oxidation and, where there is insufficient vitamin C, megaloblastic anaemia can develop.

In their cross-sectional study reported in 1988, Jacob et al. [20] investigated the relationship between vitamin C intake and folate status for an elderly population of 235 males and 442 females. They reported that the users of vitamin C supplements had a 25% higher plasma folate concentration than those who did not use the supplements.

In 2002, Cafolla et al. [21] reported on their randomised controlled trial to investigate the effect of vitamin C and folic acid supplements on red-cell folate, serum folate and serum homocysteine concentrations in Italian blood donors who smoked. The 100 participants were randomly divided equally into four groups, covering all combinations of treatment or no treatment of each of the two vitamins. Red-cell folate and serum folate concentrations increased significantly in those taking vitamin C alone; the increase was much greater in those taking folic acid alone, and highest in those taking both supplements; there was no increase in those taking no supplements. These results are consistent with those found in previous studies. Homocysteine concentrations decreased in those taking folic acid alone; the decrease was less for those taking both supplements, and concentrations increased significantly for those on vitamin C alone. Cafolla et al. proposed that these results could be explained by the high doses of vitamin C interfering with vitamin B_12_ absorption but did not provide any vitamin B_12_ data to support this hypothesis.

In their report of 2008, Verlinde et al. [22] described an interventional study of the short-term effect of simultaneous intake of vitamin C and a natural folate derivative, (6S)-5-Methyltetrahydrofolic acid, on 9 healthy adult male volunteers. They reported a significant improvement in serum folate response to the folate intake when vitamin C was included but cautioned against extrapolation of their results to long-term folate status. They noted that their results were consistent with those reported by Cafolla et al. [21] who used folic acid supplements rather than natural folate.

In their cross-sectional study of 212 adults, reported in 2012, Lucock et al. [23] investigated the relationship between vitamin C intake and folate status. They reported that, at high intakes of natural folate (5-methyltetrahydrofolate, 5-methylTHF, 5CH_3_-H_4_-PteGlu_n_), red-cell folate correlates with the level of intake of both vitamin C and folate. When the folate was in the synthetic folic acid form (pteroylglutamic acid, PteGlu), red-cell folate was correlated with the level of vitamin C intake but not with the level of folate intake. They also reported that some folate gene expressions were influenced by the combination of vitamin C and natural folate intakes. The authors suggest that taking vitamin C with natural folate may replace the potentially harmful synthetic form of folic acid currently used for supplementation.

### Proposed mechanisms for the influence of vitamin C status on time taken to develop folate deficiency

**Fig. 4:**
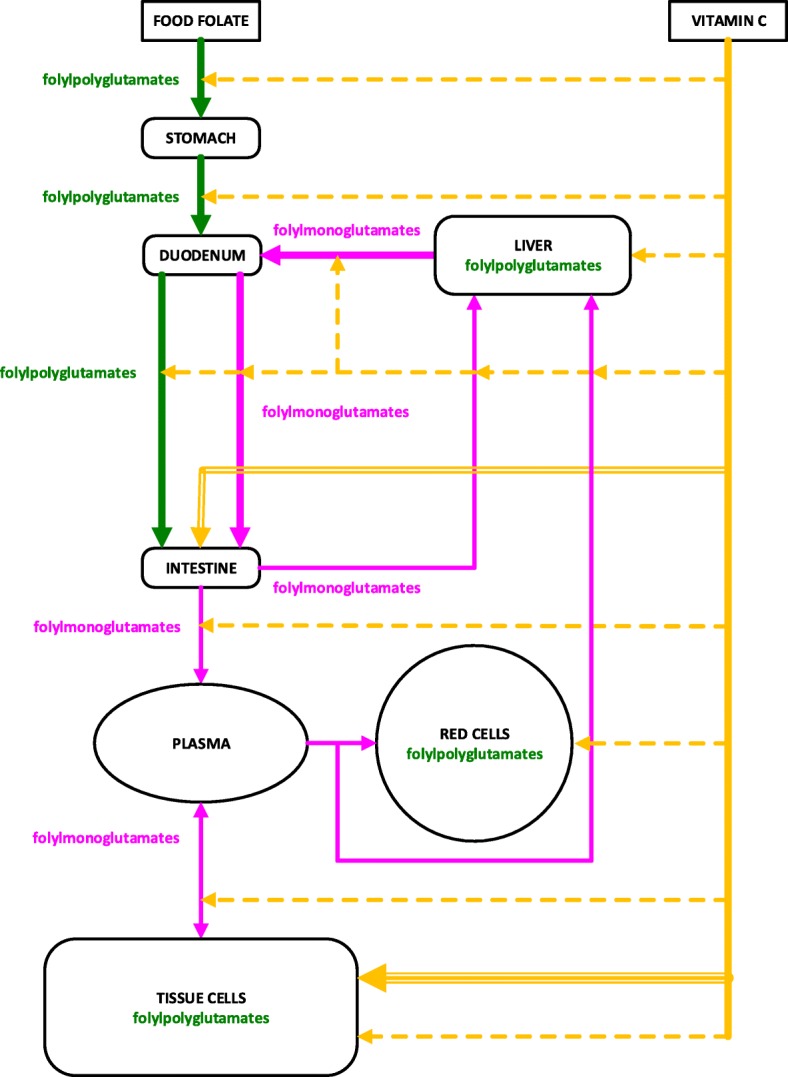
Proposed mechanisms for the influence of vitamin C status on time taken to develop folate deficiency. The double orange line indicates the site for the first proposed mechanism for the influence of vitamin C status on time taken to develop folate deficiency; vitamin C assists in the deconjugation of folylpolyglutamates to folylmonoglutamates in the intestine. The triple orange line indicates the site for the second proposed mechanism; the conversion of 5-Methyldihydrofolic acid into bio-active 5-Methyltetrahydrofolic acid in tissue cells. The dashed orange lines indicate the sites for the third proposed mechanism; reduced folates are protected from oxidation by vitamin C

In-vitro experiments have demonstrated that vitamin C assists deconjugation of polyglutamates to the monoglutamate form, converts 5-Methyldihydrofolic acid into 5-Methyltetrahydrofolic acid and protects reduced folates from oxidation. These three proposed mechanisms, by which vitamin C affects the time taken to develop folate deficiency, are illustrated in Fig. 4. The findings of the in-vivo experiments of Cafolla et al. [21] and Verlinde et al. [22] were consistent with these in-vitro models.

In 2008, Verlinde et al. [22]. reviewed the mechanisms of transport and absorption of folates, and the influence of vitamin C intake on folate status. Background to their study were two mechanisms by which vitamin C has been found to affect folate status in-vitro. Firstly, they cite Donaldson and Keresztesy [24], and Lucock et al. [25], who found that vitamin C converts 5-Methyldihydrofolic acid into bio-active 5-Methyltetrahydrofolic acid. Secondly, they cite Oey et al. [26] who found that (6S)-5-Methyltetrahydrofolic acid is protected from oxidation by vitamin C. They then cite Lucock et al. [25] and Thien et al. [27] who proposed that vitamin C could significantly affect folate bioavailability in-vivo; it was the lack of direct in-vivo evidence to support this hypothesis that inspired the study by Verlinde et al.

In 2017, Ringling and Rychlik [28] experimentally investigated folate bioavailability of folates in several foods by using an in-vitro bio-accessibility model of the gastro-intestinal digestion of folate. The model confirmed that vitamin C increases folate bioavailability by protecting methyltetrahydrofolic acid from oxidation. The model also supported the proposal that vitamin C increases the efficiency of conversion of polyglutamates, in food, to the monoglutamate form required for absorption in the small intestine. As discussed by McNulty and Pentieva [29], whether or not monoglutamates are more bio-available than polyglutamates is controversial.

### The sigmoidal relationship between vitamin C intake and plasma concentration

Cross-sectional studies have revealed a sigmoidal relationship between plasma concentration of vitamin C and vitamin C intake. In their cross-sectional study of an elderly population of 138 males and 166 females on high vitamin C intakes, Garry et al. [30] found that plasma vitamin C concentration increased rapidly up to a daily vitamin C dose of 150 mg, then the rate of increase slowed significantly. Newton et al. [31] also reported a sigmodal relationship between plasma concentration of vitamin C and vitamin C intake in their study of 101 elderly women on lower vitamin C intakes. Jacob et al. [20] also plotted plasma vitamin C concentration against the daily vitamin C dose, for an elderly population of 235 males and 442 females. This again produced a sigmoid curve, with plasma concentration of vitamin C saturating, approaching a plateau of 90 μmol/L for males and 100 μmol/L for females.

### Should vitamin C intake be sufficient to ensure plasma/tissue saturation?

Historically, there has been no consensus on the optimal human vitamin C intake and whether or not the intake should be sufficient to ensure plasma/tissue saturation (Table 1).

**Table 1:** Recommended daily intake of vitamin C for adults

Reference	Male RDI mg/day	Female RDI mg/day	Plasma concentration μmol/L	Criteria
Garry et al. [30]	150 (elderly men)	75 (elderly women)	57^c^	*“If maintaining a maximum body pool is a desirable end point”*
Newton et al. [31]	N/A	60 (elderly women)	20	*“ensure against impairment of health”*
Olson and Hodges [33]	40	30	14^b^	Scurvy - *“To maintain a suitable body pool”*
Levine et al. [32]	200	200	66^a^	*“first dose beyond sigmoid part”*
Carr and Frei [35]	120	120	58^b^	*“reduced risk of cardiovascular disease and cancer”*
Institute of Medicine [38]	90	75	48^b^	*“80% of maximal neutrophil concentration”*
FAO/WHO [39]	45	45	17^b^	*“A body content of 900 mg falls half way between tissue saturation and the point at which clinical signs of scurvy appear”*
				*“45 mg would achieve 50% saturation in the tissues”*
Levine et al. [48]	N/A	90 (healthy young women)	48^b^	*“using Food and Nutrition Board guidelines”*
Carr et al. [34]	120–200	120–200	58-66^a^	*“optimal intake of vitamin C required to maintain general health and wellbeing”*
NHMRC [40]	45	45	17^b^	*“body content is halfway between tissue saturation and the point at which clinical signs of scurvy appear”*
UK Government [49]	40	40	14^b^	Not specified in reference

^a^mean of 7 patients, derived from Levine et al. [32]

^b^estimated from Fig. 5 and table and chart in Additional file 2

^c^converted from stated concentration of 1.0 mg/dL to SI units using formula provided by Newton et al. [31]: 1.0 μmol/L ≈ 17.6 μg/100 mL

Based on the results of their 1996 longitudinal study of 7 healthy volunteers, Levine et al. [32] recommend 200 mg vitamin C daily; a level that ensures plasma saturation. They plotted plasma vitamin C concentration against the daily vitamin C dose; this produced a sigmoid curve with 200 mg the lowest daily dose to reach a plateau. They also noted that the current RDA is inadequate because this intake is on the rapidly rising slope, so plasma vitamin C concentration would be very sensitive to variations in intake.

As an example of earlier opinion, Olson and Hodges [33] in 1987 recommend only 40 mg/day for men and 30 mg/day for women. They dispute the need to achieve tissue saturation and state that, in such a saturated condition, the body excretes excess vitamin C to control the blood concentration. The 40 mg/day dose would produce a plasma concentration of only 14 μmol/L, compared to the 66 μmol/L for the 200 mg/day intake recommended by Carr et al. [34] and Levine et al. [32].

As commented by Carr and Frei in their 1999 review [35], and Levine and Eck in their 2009 editorial [36], current official Recommended Daily Intakes (RDIs) or Recommended Dietary Allowances (RDAs) are designed only to prevent scurvy. Such vitamin C intakes are not sufficient to ensure tissue saturation, whereas several researchers recommend higher intakes that will ensure tissue saturation. Carr and Frei recommend doubling the RDA for vitamin C from 60 mg/day to 120 mg/day for healthy non-smoking adults.

Recent studies support a vitamin C intake that ensures plasma saturation. In 2012, Carr et al. [34] recommend increasing the RDI for vitamin C, to 120–200 mg/d, to ensure that the plasma is saturated, at a concentration of 70 μmol/L. In 2010 Lykkesfeldt and Poulsen [37] stated that, although defining an optimal intake of vitamin C is controversial, there is now general agreement that it should be sufficient to ensure plasma saturation.

### Current official recommended vitamin C intakes

The Institute of Medicine (US) Panel on Dietary Antioxidants and Related Compounds [38] recommends 90 mg/day for men and 75 mg/day for non-pregnant women, citing Levine et al. [32] as their data source. Based on their criteria of providing just sufficient vitamin C to efficiently work as an antioxidant, they calculated a dose that ensures a midpoint between 60 and 100% of maximum neutrophil saturation. Although the authors discuss the protection of low-density lipoprotein (LDL) and intracellular proteins from oxidation, they do not specifically consider the level of vitamin C intake required to protect folates.

The FAO/WHO expert consultation on human vitamin and mineral requirements [39] recommends a vitamin C intake of 45 mg/day. This intake is based on their calculation for a dose that maintains a body store of 900 mg, being half-way between that required to avoid scurvy and 100% tissue saturation; the report notes that this level of intake produces a plasma concentration low on the slope of the intake-plasma concentration curve. This conflicts with the recommendation of Levine et al. [32] for an intake that ensures tissue saturation i.e. a dose on the plateau of the curve.

The Australian Government National Health and Medical Research Council (NHMRC) [40] recommends a vitamin C RDI of 45 mg/day for men and non-pregnant women, based on the same criteria as used by the FAO/WHO [39]. According to Carr et al. [34], the resulting plasma concentration of < 23 μmol/L is too low.

### Comparison of vitamin C status for Herbert and this author

**Fig. 5:**
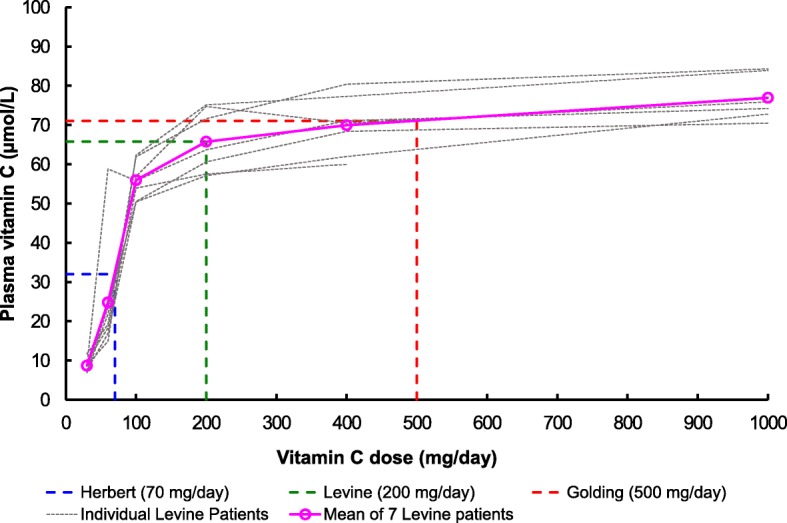
Plasma vitamin C concentration vs vitamin C dose. Derived from Levine et al. [32]

Herbert [1] consumed 70 mg/day of vitamin C during his self-experiment, whereas this author [10] consumed 500 mg/day. This author used data from Levine et al. [32] to produce a chart (Fig. 5) to illustrate the relationship between vitamin C dose and the plasma concentration, at the levels relevant to this discussion.

At the dose of 200 mg/day recommended by Levine et al. [32], the plasma concentration is 66 μmol/L. At this author’s dose of 500 mg/day, the plasma concentration is 71 μmol/L, very close to the 70 μmol/L considered by Carr et al. [34] to be necessary for good health, and on the plateau of the dose/plasma concentration curve, as recommended by Levine et al.

At Herbert’s dose of 70 mg/day [1], the plasma concentration is 32 μmol/L, very significantly lower than that recommended by Levine et al. [32]. Also, Herbert’s vitamin C intake places his plasma concentration on the rapidly rising part of the curve and, as noted by Levine et al., any error in the intake will cause a proportionately larger error in the plasma concentration.

Another factor to be considered, when comparing the vitamin C status for the subjects of the two self-experiments, is the potential effect of the iron supplements on the amount of vitamin C available to protect the reduced folates. The iron tablets taken by this author (Abbott Australia Ferro-Grad C) comprised 325 mg ferrous sulphate (equivalent to 105 mg elemental iron), with 562 mg sodium ascorbate (equivalent to 500 mg vitamin C). Herbert took 300 mg ferrous sulphate for 10 days from week 16, after suspecting iron deficiency, but did not increase his vitamin C intake from the 70 mg/day he had been taking.

The vitamin C, either integrated with the iron supplements in the form of sodium ascorbate or taken separately, is used to assist with absorption of the iron. From their 1960s experiments on adult males, Mc Curdy and Derne [41] found that, for vitamin C doses of up to 500 mg, absorption of ferrous sulphate increases with increased vitamin C intake. This cannot be explained by any action of vitamin C reducing the ferric form to the ferrous form because, in all of these cases (Herbert, Golding, McCurdy and Derne), the iron supplements were in the ferrous form.

Other studies confirmed that vitamin C is involved in the absorption of iron. In 1991, Siegenberg et al. [42] reported that, in their interventional study of 199 subjects, they found that vitamin C aids iron absorption by overcoming the impeding effects of phytates and polyphenols. Also in 1991, Hoffman et al. [43] described an in-vitro study in which they found that vitamin C assists in gastrointestinal absorption of iron and increases its bioavailability; although they provided a detailed discussion of the subject, they stated that the exact mechanisms by which this is achieved were not yet fully understood. In their 2007 review, Sharp and Srai [44] discuss the role of vitamin C in overcoming the impeding effects of phytates and polyphenols, but do not explain the mechanism by which this is achieved.

In 2004 Fisher and Naughton [45] discussed the potential for harmful oxidative stress caused by taking ferrous iron supplements with vitamin C. They provide details of three proposed mechanisms by which vitamin C is oxidised by reaction with ferrous iron: Udenfriend’s system; Fenton chemistry; the Weissberger system.

Some of the vitamin C supplement would have been oxidised, or otherwise chemically altered, in its role of assisting with iron absorption, and would therefore not be available to protect folates from oxidation. This effect is likely to be greater in the case of Herbert than in the case of this author because of the difference in vitamin C intake. With his dose on the plateau of the sigmoid curve, this author’s plasma vitamin C concentration would probably have been almost unaffected by the need to assist with absorption of the iron. With his dose on the rapidly rising sigmoid part of the curve, Herbert’s plasma vitamin C concentration could have been significantly decreased by the need to assist with absorption of the iron.

### Vitamin C status as potential confounding factor for Herbert and this author

If the plasma vitamin C concentration is not sufficient to prevent oxidation of fully reduced folate, although sufficient to prevent scurvy, there would be sub-optimal vitamin C status. There is no published evidence that, once there is sufficient vitamin C present to ensure plasma saturation, and thereby protect the fully reduced folates, additional amounts up to 500 mg/day will affect the folate status.

The 500 mg/day vitamin C intake for this author’s self-experiment [10], ensuring plasma saturation, would therefore be expected to have protected the body store of reduced folate from oxidation. This is likely to have ensured that the time taken to develop megaloblastic anaemia of folate deficiency was not confounded by vitamin C deficiency.

The 70 mg/day vitamin C supplement taken by Herbert during his self-experiment [1], insufficient to produce plasma saturation, would not be expected to protect the reduced folate from oxidation. This is likely to have shortened the time taken to develop megaloblastic anaemia of folate deficiency, compared to the case if the same subject had taken sufficient vitamin C supplement to ensure plasma saturation.

## Conclusions

Victor Herbert developed megaloblastic anaemia four months after commencing a severely folate-deficient diet whereas, in his self-experiment 50 years later, this author took 19 months to fully deplete his liver folate store. This author now proposes that this time difference was caused not only by his own vegetarian diet and consumption of folate-fortified foods; Herbert was likely to have been deficient in vitamin C, thus shortening the time taken to develop folate deficiency.

Several human experiments have confirmed the role of vitamin C in protecting reduced forms of folate from oxidation. Although there has historically been no consensus on the required intake of vitamin C, and official recommendations set a level below that required to ensure plasma saturation, recent research supports an intake that would ensure saturation.

Herbert’s vitamin C intake was insufficient to fully protect reduced folates from oxidation; this could have significantly shortened the time taken to develop megaloblastic anaemia of folate deficiency. In his own self-experiment, this author’s saturated vitamin C status protected his folate stores from oxidation; the liver storage time was therefore far longer.

There have been no longitudinal experiments on human subjects since the introduction of voluntary or mandatory folic acid fortification of food, and the few published models differ significantly in their estimates of human liver folate storage capacity. Because of the importance of folate in one-carbon metabolism, the potential influence of vitamin C intake on the time taken to deplete the liver folate store should be experimentally investigated.

## Additional files

Additional file 1: Microsoft Excel table and chart for Fig. 3. (XLSX 43 kb)

Additional file 2: Microsoft Excel table and chart for Fig. 5. (XLSX 21 kb)

Additional file 3: High-resolution images for Figs. 1, 2, 3, 4 and 5. (PDF 373 kb)

Additional file 4: High-resolution slides for Figs. 1, 2, 3, 4 and 5. (PPTX 1057 kb)

## Abbreviations

FAO: The Food and Agriculture Organization of the United Nations
MCV: Mean Cell Volume
RDA: Recommended Dietary Allowance
RDI: Recommended Daily Intake
WHO: World Health Organization
