# Supplementary figures and images for: Experimental folate deficiency in human subjects: what is the influence of vitamin C status on time taken to develop megaloblastic anaemia?
Source: BMC Hematol. 2018 Jun 19;18:13. doi: 10.1186/s12878-018-0107-2 (PMC6007024; doi:10.1186/s12878-018-0107-2)

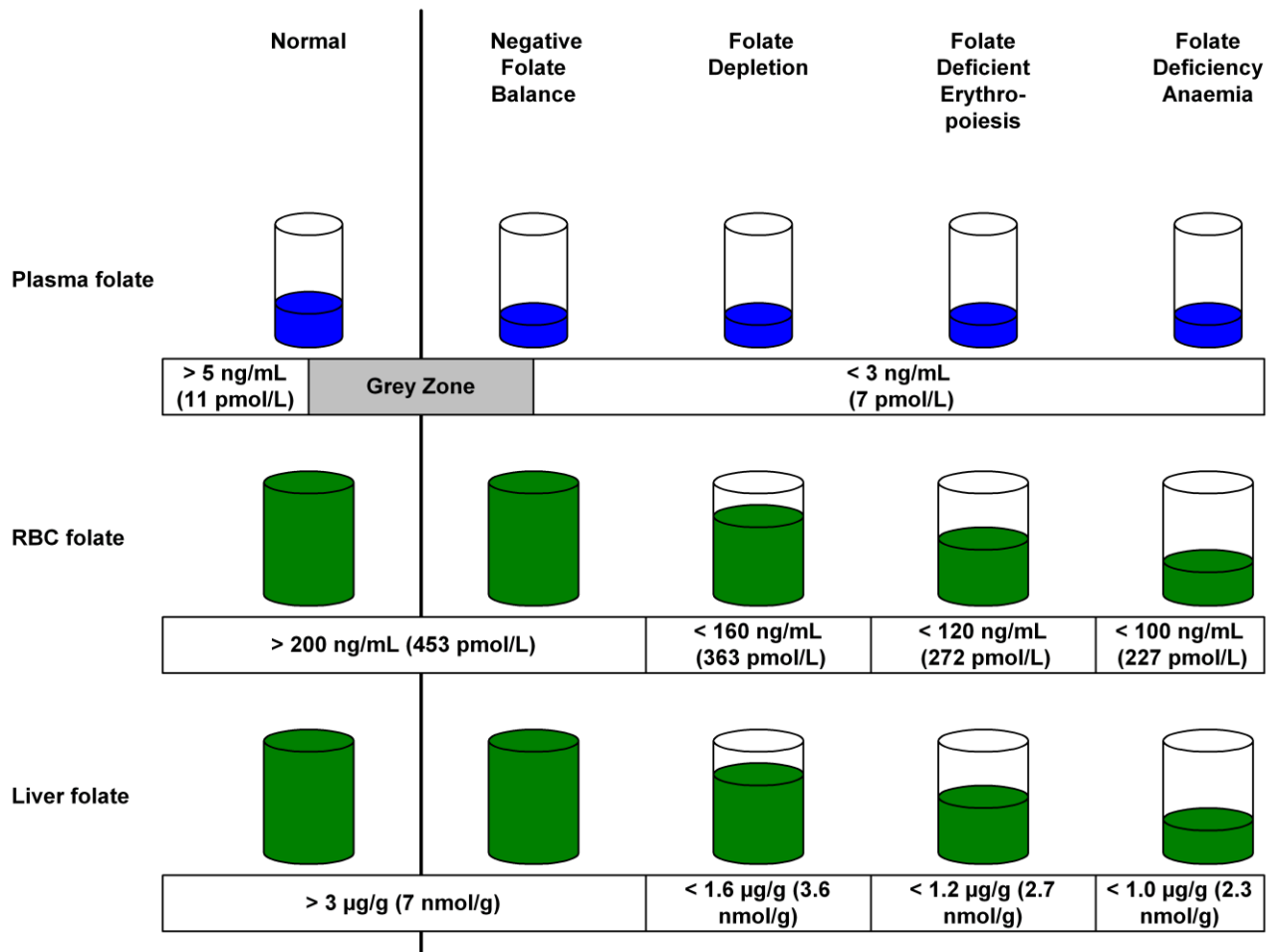

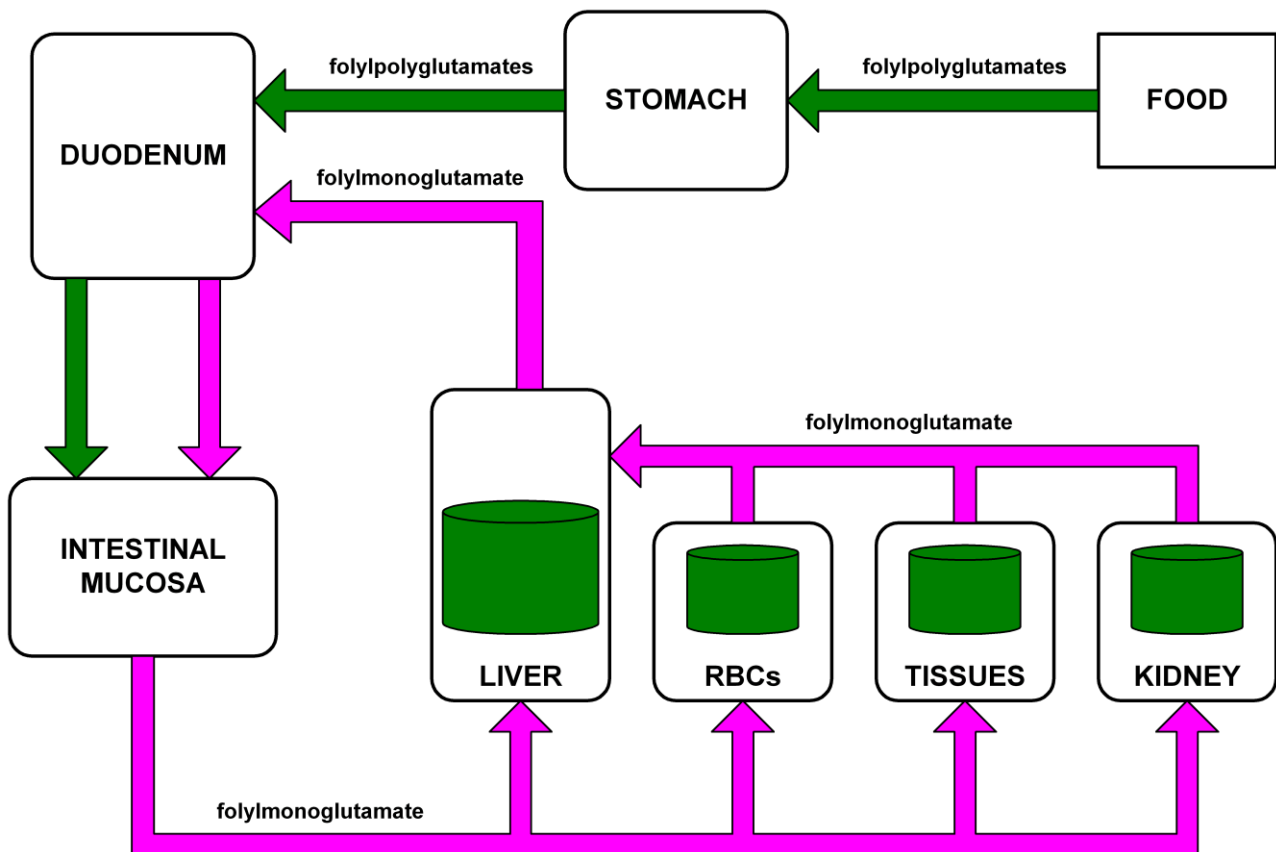

## A Serum folate

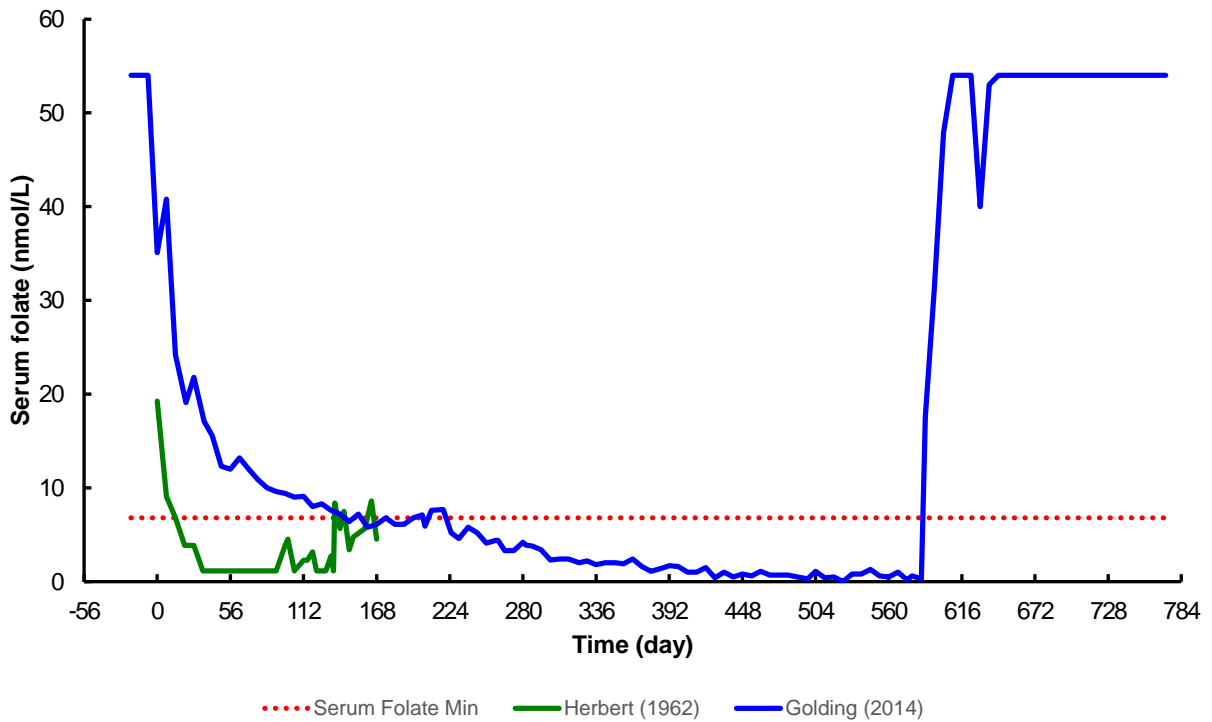

## B Red-cell folate

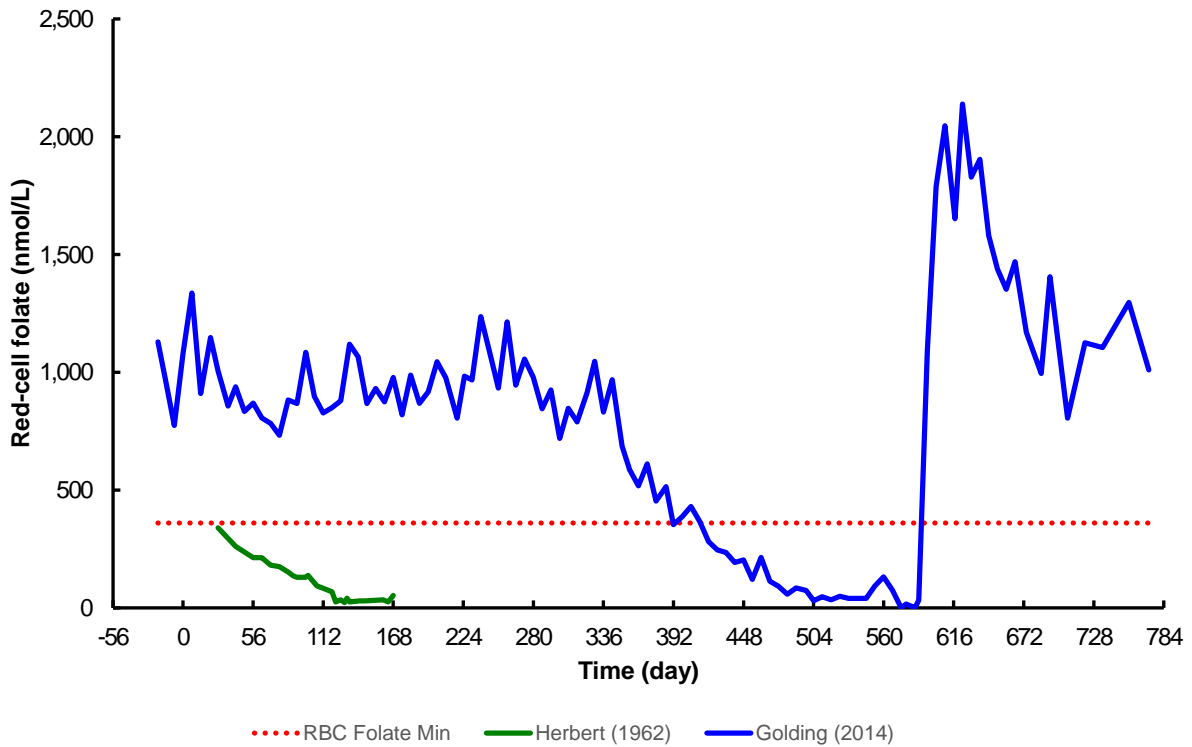

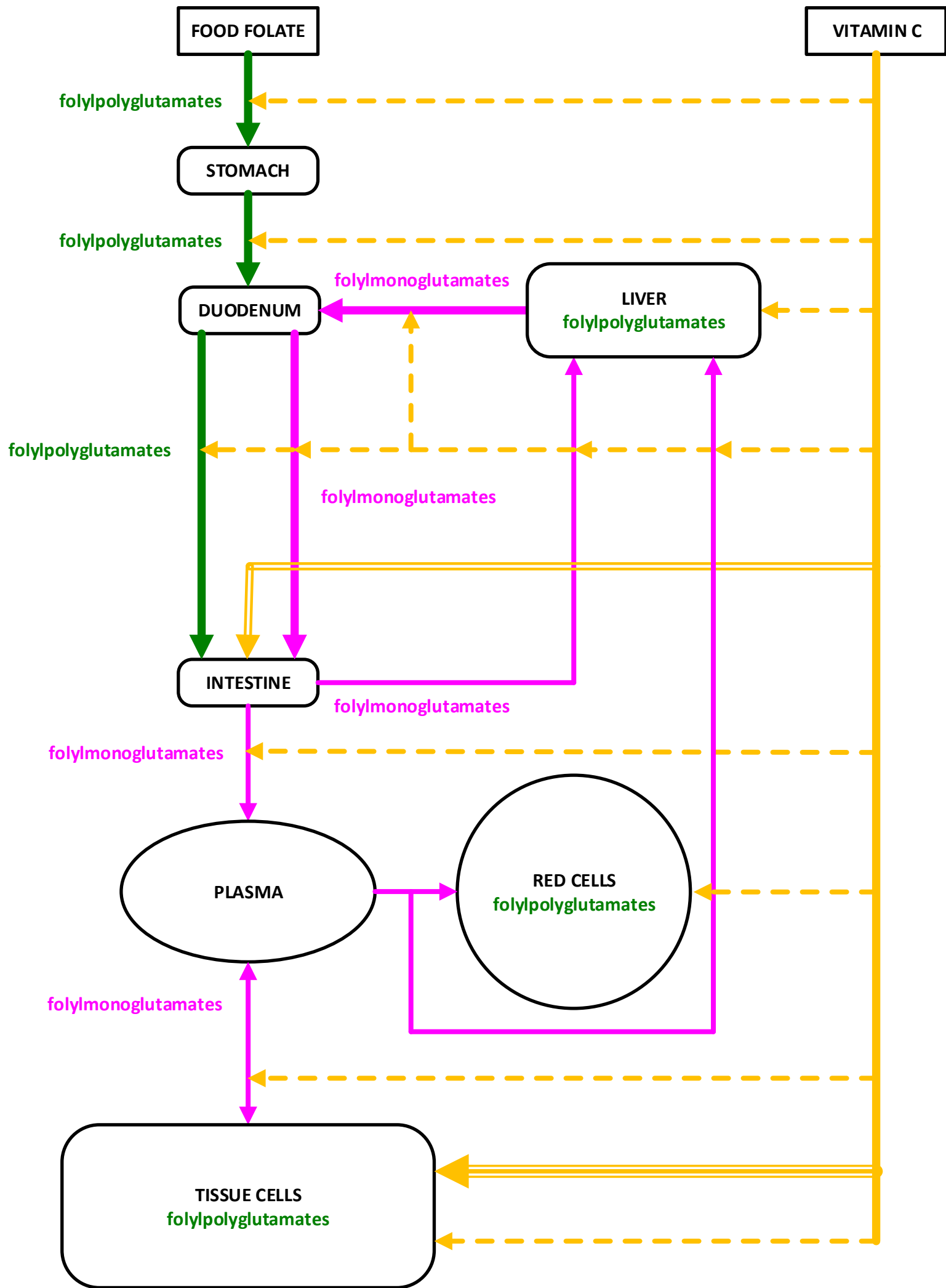

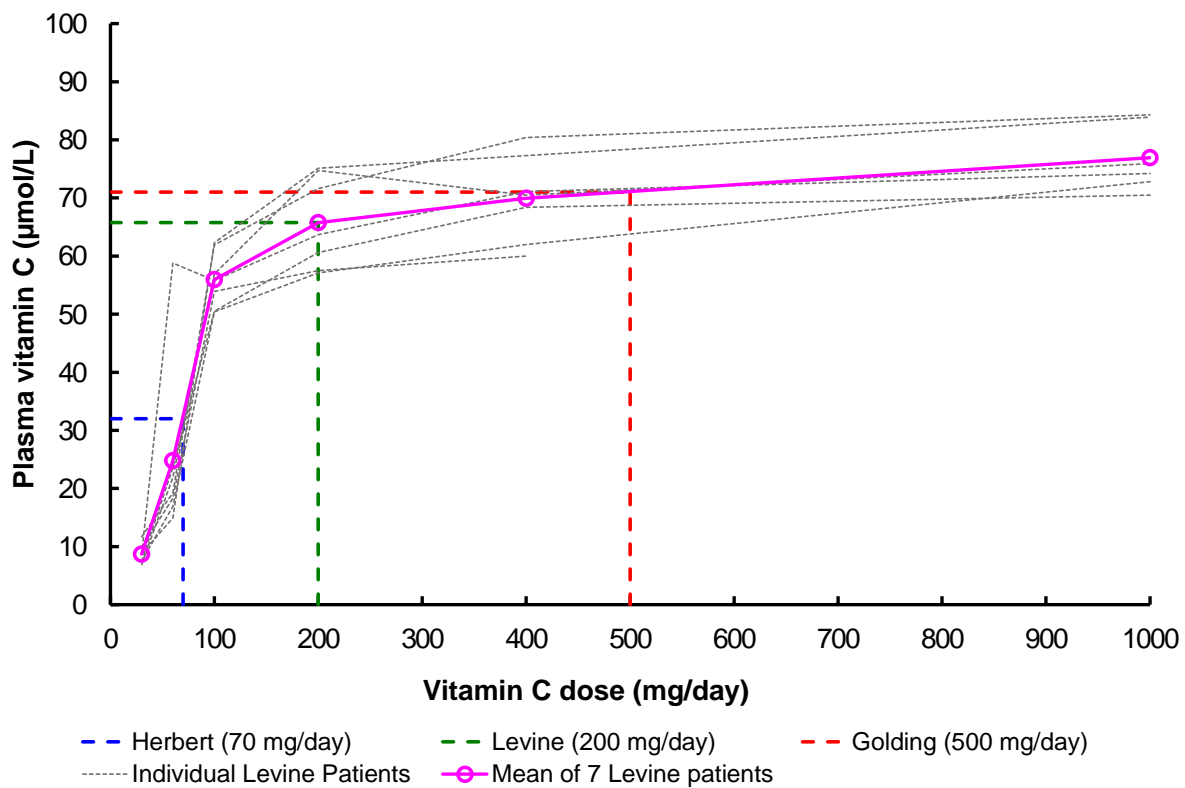

Supplement: Supplementary file 3 — High-resolution images for Figs. 1, 2, 3, 4 and 5. (PDF 373 kb) [file 12878_2018_107_MOESM3_ESM.pdf]

## Slide 1
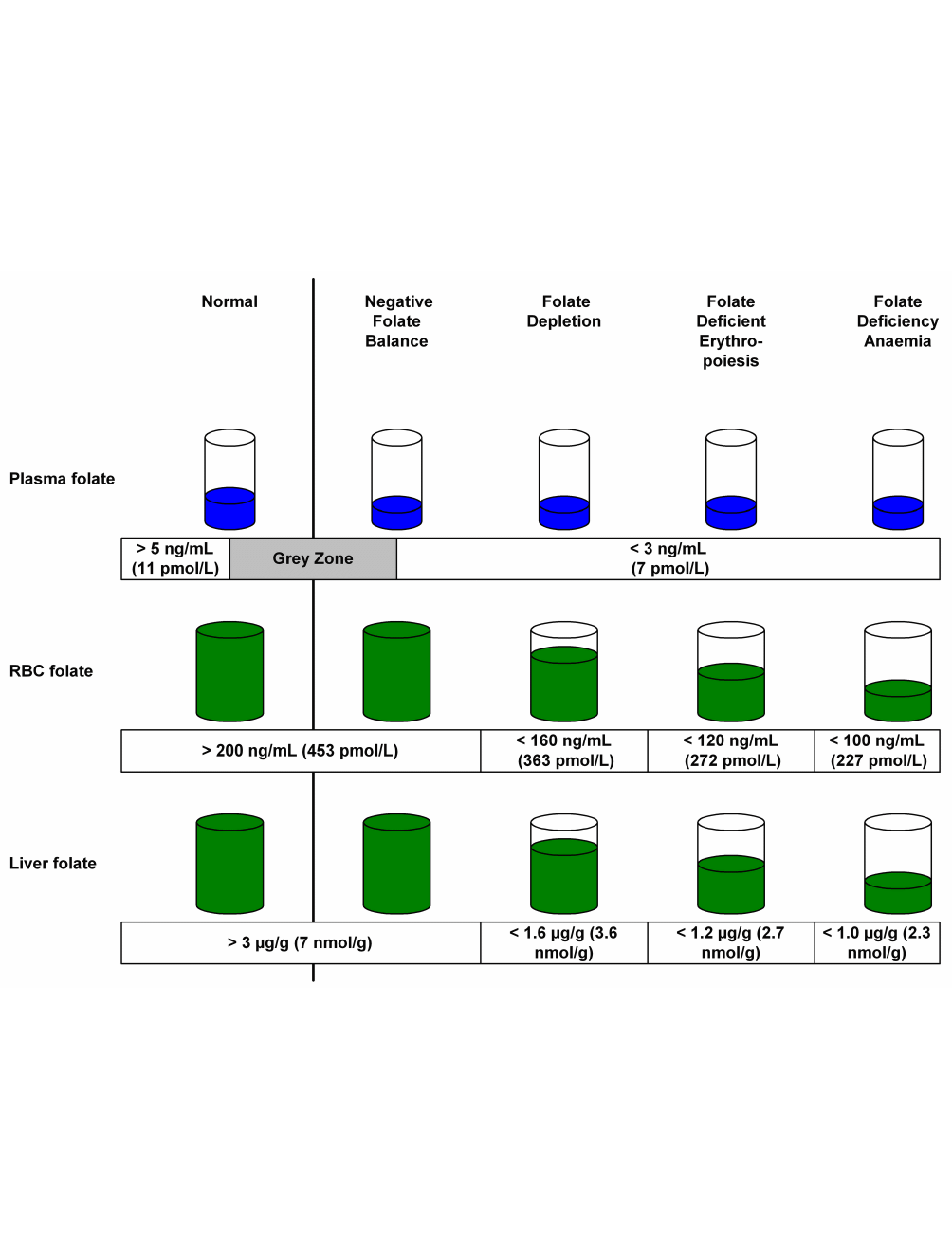

## Slide 2
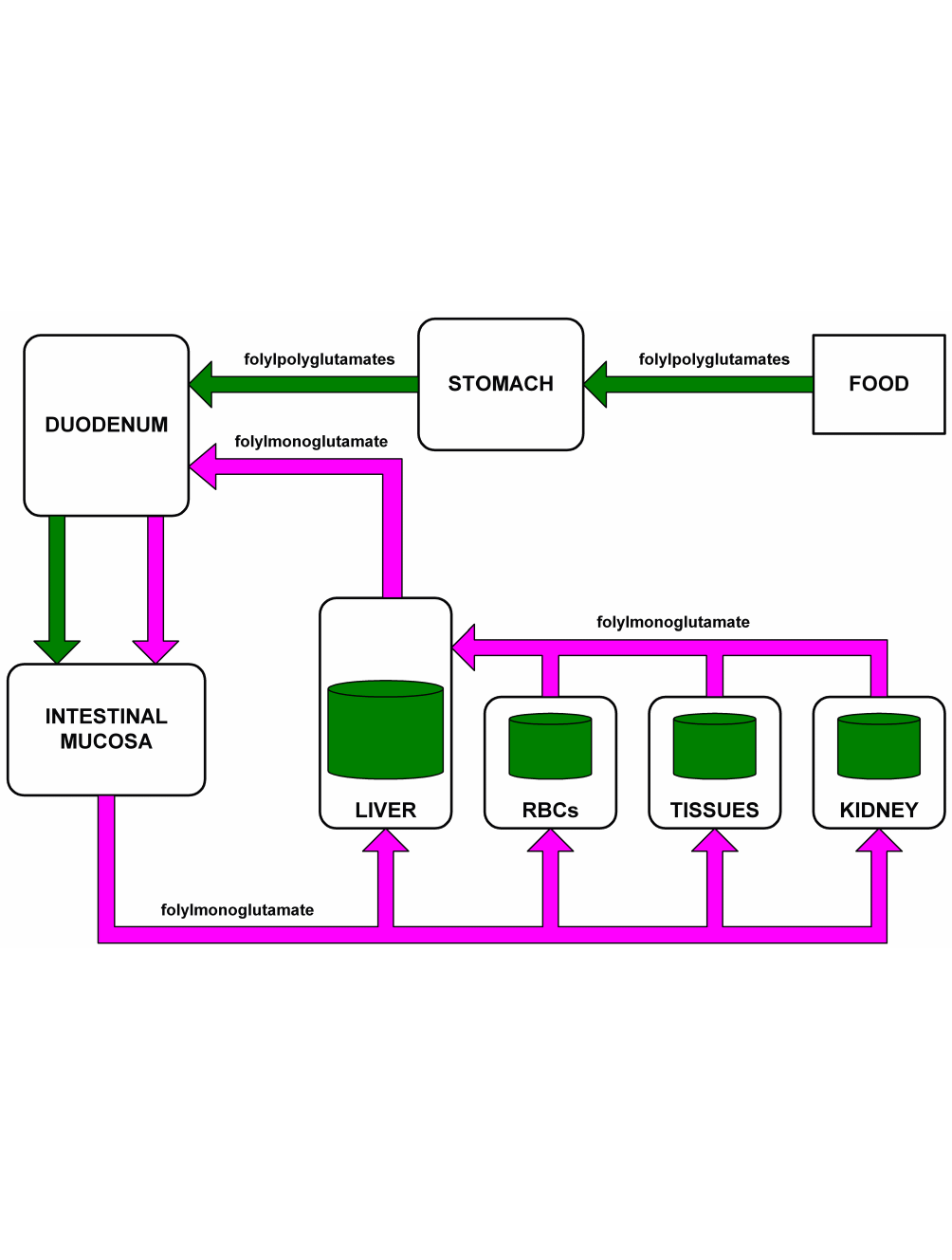

## Slide 3
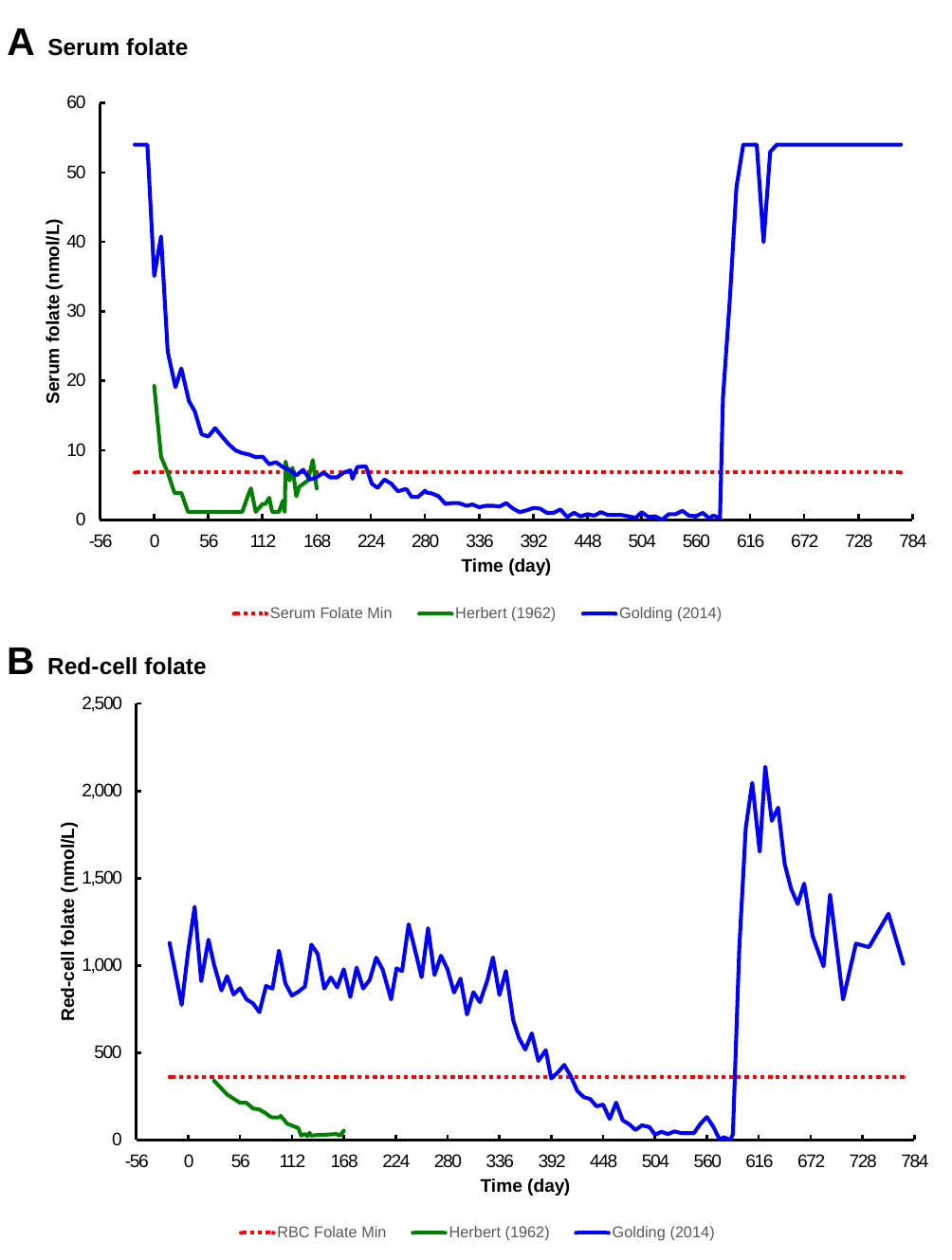

## Slide 4
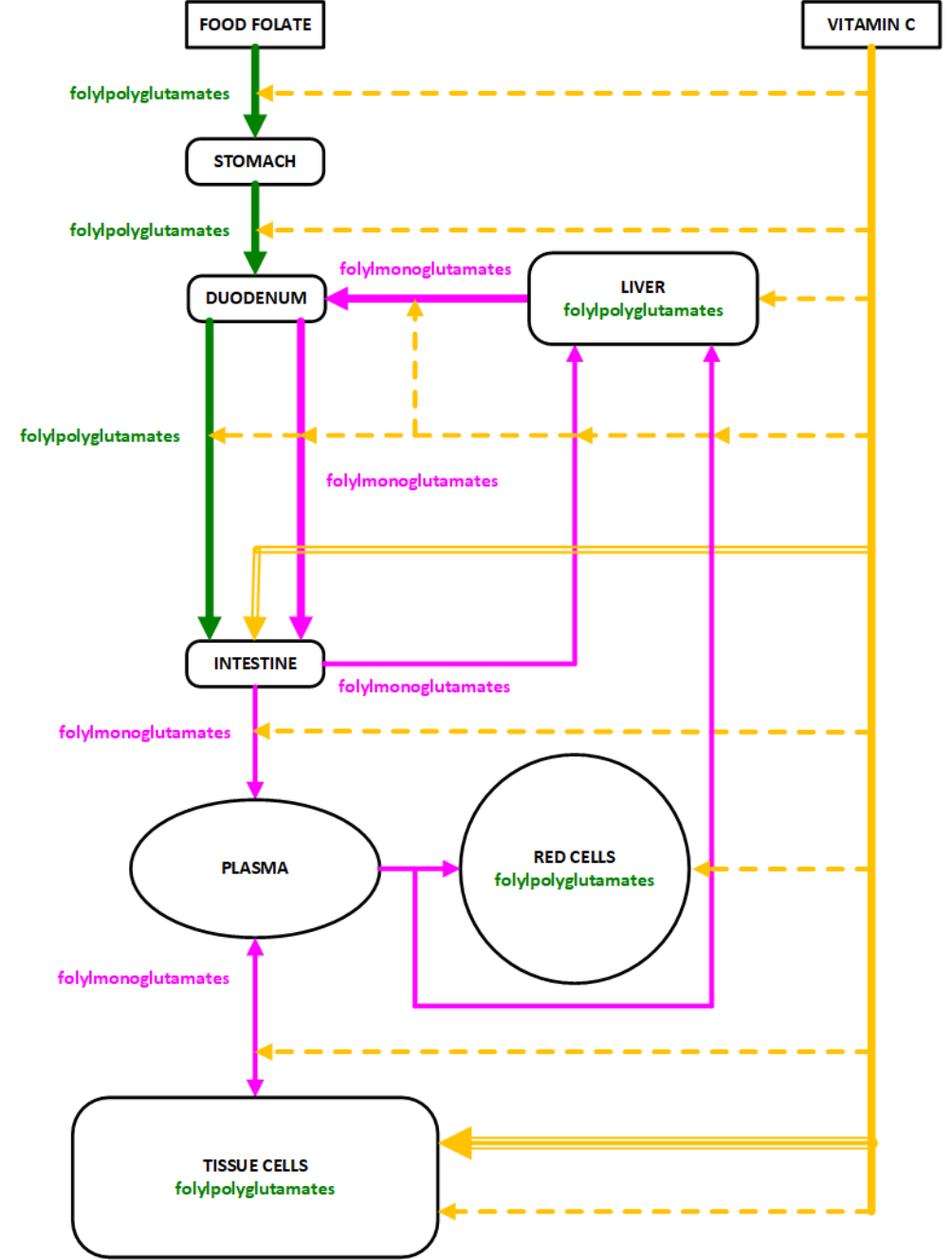

## Slide 5
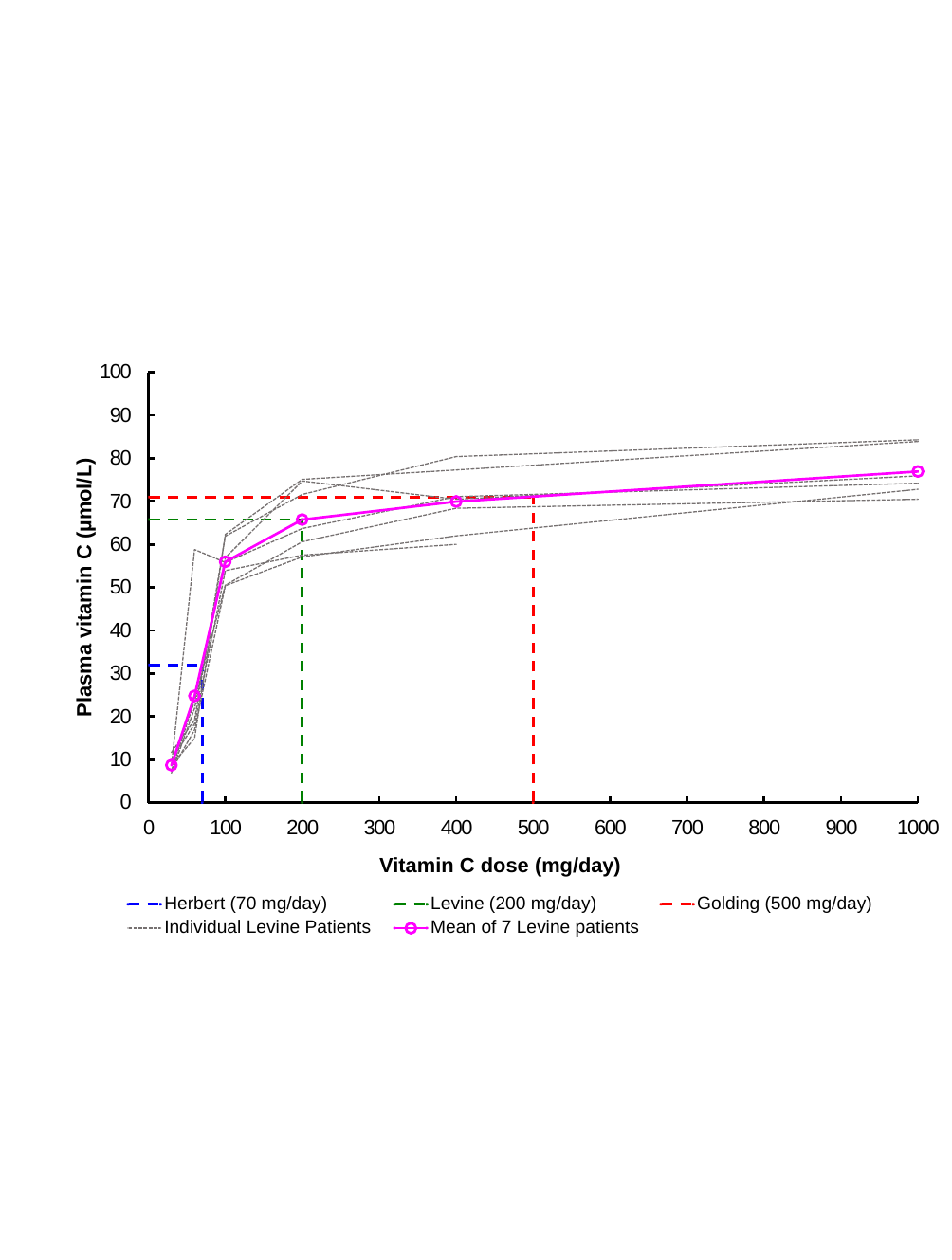

Supplement: Supplementary file 4 — High-resolution slides for Figs. 1, 2, 3, 4 and 5. (PPTX 1057 kb) [file 12878_2018_107_MOESM4_ESM.pptx]
